# Evaluation of cell surface vimentin positive circulating tumor cells as a prognostic biomarker for stage III/IV colorectal cancer

**DOI:** 10.1038/s41598-023-45951-1

**Published:** 2023-11-01

**Authors:** Jiazi Yu, Mian Yang, Tao Peng, Yelei Liu, Yuepeng Cao

**Affiliations:** 1 Department of Colorectal Surgery, Ningbo Medical Centre Li Huili Hospital, Ningbo, People’s Republic of China; 2 Department of General Surgery, Ningbo Medical Treatment Centre Li Huili Hospital, 1111 Jiangnan Road, Ningbo, 315000 People’s Republic of China; 3 Department of Colorectal Surgery, The First Affiliated Hospital of Ningbo University, Ningbo, People’s Republic of China

**Keywords:** Cancer models, Cancer therapy, Metastasis, Tumour biomarkers

## Abstract

Currently, little is known about the phenotypes of circulating tumor cells (CTCs), particularly epithelial and mesenchymal phenotypes, and their impact on the prognosis of colorectal cancer (CRC) patients. This study aims to investigate the CTC phenotypes and their prognostic implications in stage III/IV CRC. Patients who were diagnosed with CRC and underwent CTC detection at two hospitals were included. CTCs were detected using a mesenchymal CTC kit, and the clinical and pathological characteristics of CTCs were compared with those of cell surface vimentin-positive CTCs (CSV-CTCs). Disease-free survival (DFS) was assessed and used as an indicator of CTC phenotype-related prognosis. Univariate and multivariate Cox regression analyses were made to identify risk factors, and nomogram models were employed for prognostic prediction. A total of 82 patients were enrolled, with a CTC detection rate of 86.6%. Among the detected CTCs, 60% were CSV-CTCs. The CSV-CTC count showed a positive correlation with the T-stage, the M-stage, and the location of the primary tumor (P = 0.01, P = 0.014, and P = 0.01, respectively). Kaplan–Meier survival analysis revealed that CSV-CTCs were associated with worse DFS in patients receiving first-line oxaliplatin chemotherapy (hazard ratio (HR) = 3.78, 95% CI 1.55–9.26, p = 0.04). When the cut-off value of the CSV-CTC count was 3, the optimal prognostic prediction was achieved. Compound models considering CSV-CTCs, TNM staging, the site of the primary tumor and the Ras gene status yielded the best results in both the receiver operating characteristic (ROC) analysis and the decision curve analysis (DCA). This study indicates that CSV-CTCs predominate in CTCs of CRC patients, and a count of CSV-CTCs ≥ 3 is an independent risk factor for worse prognosis.

## Introduction

In 2018, CRC ranked 2nd in prevalence and 3rd in cancer-related deaths worldwide^1^. In China, the incidence and mortality of CRC have increased steadily since 2000. In 2015, there were 376,300 new cases and 191,000 deaths^2^. In spite of recent advances in diagnostic modalities, a significant proportion of patients are diagnosed with advanced cancer (2). These patients are still at high risk of tumor recurrence and metastasis even after radical resection and adjuvant chemotherapy^3^. The use of predictive biomarkers and an understanding of their responses are crucial to optimizing CRC treatment^4–6^.

CTCs are shed from the primary tumor and distributed into the blood. They have been studied extensively over the past decade as predictors of prognosis and responses to therapy. A common method of detecting CTCs is to use the epithelial cell adhesion molecule (EpCAM), an epithelial cell marker^7^. Despite being epithelial in origin, CTCs still undergo an epithelial-mesenchymal transition (EMT). As a result, they acquire mesenchymal characteristics and become more motile and invasive^8,9^. There has been a logical conflict for years between EpCAM-based CTC identification and EMT theory. In our previous study^10^, we applied the CellRich™ platform to the detection of epithelial CTCs. The study results demonstrated that the CTC count in patients with stage III/IV CRC could be used to predict the efficacy of platinum-based first-line chemotherapy. High expression of vimentin (an indicator of EMT) in tumor tissue biopsies was identified as an independent risk factor for a high CTC count in peripheral blood. High counts of CTCs may be associated with EMT. For these reasons, we assumed that CTCs were theoretically more likely to have a mesenchymal phenotype. The use of EpCAM could result in an underestimation of the CTC count as EpCAM-negative cells were excluded, which might have a mesenchymal phenotype and played a crucial role in distant metastasis. Even though a previous study has investigated different phenotypes of CTCs in CRC patients^11^, there is still a lack of research on how these varied CTC types affect patient prognosis.

In addition to the analysis of epithelial and mixed CTCs, we utilized the CytoSorter^®^ CSV mesenchymal CTC kit (Hangzhou Watson Biotech, Hangzhou, China) to identify and enrich CTCs expressing cell-surface vimentin, and assessed the clinical significance of their presence in CRC patients in the present study. Furthermore, to facilitate clinical application, we developed nomogram models.

## Results

### Clinicopathological features of CRC patients

A total of 82 patients (38 males and 44 females) were enrolled, with 42 patients from Ningbo Medical Center Li Huili Hospital and the other 40 patients from the First Afflicted Hospital of Ningbo University. The demographic and clinicopathological characteristics of the patients are presented in Table 1. No significant differences were observed between the two cohorts. Among patients with stage IV CRC, 18 had liver metastasis and 5 had lung metastasis.

**Table 1:** Characteristic of patients in Ningbo Medical Center Li Huili Hospital and The First Afflicted Hospital of Ningbo University.

Characteristic		LHL hospital (n = 42)	The first hospital (n = 40)	p-value
Age, n (%)	< 60	14 (33.3)	15 (37.5)	0.89
	≥ 60	28 (69.7)	25 (62.5)	
Gender, n (%)	Male	22 (52.4)	15 (37.5)	0.18
	Female	20 (47.6)	25 (62.5)	
T-Stage, n (%)	T1-2	2 (4.8)	1 (2.5)	0.82
	T3	14 (33.3)	14 (35.0)	
	T4	26 (61.9)	30 (75.0)	
N-Stage, n (%)	N1	19 (45.2)	10 (25.0)	0.55
	N2	23 (54.8)	26 (65.0)	
M-Stage, n (%)	M0	29 (69.0)	22 (68.8)	0.11
	M1	13 (31.0)	10 (31.2)	
CEA level, n (%)	Normal	17 (40.5)	22 (55.0)	0.19
	Abnormal	25 (59.5)	18 (45.0)	
Tumor location, n (%)	Right	15 (35.7)	11 (25.5)	0.15
	Left	27 (64.3)	29 (74.5)	
BRAF gene status	Wild	37 (88.1)	35 (87.5)	0.89
	Mutation	5 (11.9)	5 (22.5)	
RAS gene status	Wild	28 (66.7)	22 (55.0)	0.28
	Mutation	14 (33.3)	18 (45.0)	

*CEA* Carcinoembryonic antigen.

LHL hospital: Ningbo Medical Center Li Huili Hospital.

The first hospital: The First Afflicted Hospital of Ningbo University.

### Evaluation of CSV-CTCs in patients with stage III/IV CRC and their relationship with clinicopathological characteristics

**Figure 1:**
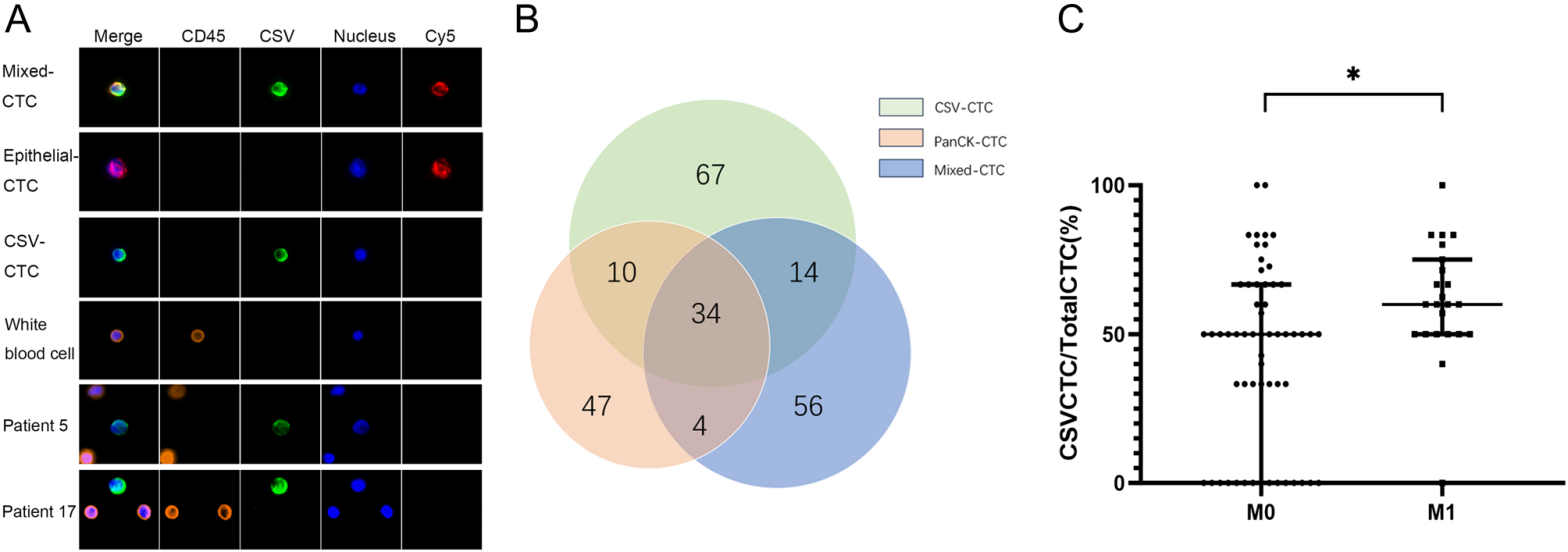
CTC phenotypes in stage III/IV CRC patients. (**A**) Immunofluorescent staining of a captured CSV-CTC and a white blood cell. (**B**) A Venn diagram of the number of patients with 1, 2, or 3 different CTC phenotypes. (**C**) The proportion of CSV-CTCs among total CTCs in stage III and IV CRC patients. “*”indicate 0.01 < P < 0.05.

Three distinct phenotypes of CTCs were identified in patients with stage III/IV CRC, namely CSV-CTCs (vimentin + CD45-PanCK-), PanCK-CTCs (vimentin- CD45-PanCK +), and Mixed-CTCs (vimentin + CD45-PanCK +) (Fig. 1A). CTCs were detected in the peripheral blood of 71 out of 82 patients, representing a detection rate of 86.6%. The median CTC count of 82 CRC patients was 4 (range: 0–11). The median CTC count of the 71 patients with positive CTC expression was 5, including 3 CSV-CTCs, 1 PanCK-CTC and 1 Mixed-CTC. A Venn diagram illustrated the number of patients with 1, 2, or 3 different CTC phenotypes (Fig. 1B). In the 59 patients diagnosed with stage III CRC, the mean and median CTC counts were 3.3 ± 2.4 and 3, respectively. The mean and median CTC counts in the 23 patients with stage IV CRC were 5.3 ± 2.1 and 5, respectively. Patients with stage IV CRC had a significantly higher count of CTCs than patients with stage III CRC (t = 3.39, P < 0.01), and the CTCs in the former were mostly CSV-CTCs (Table 2). By comparing CTC counts with other clinicopathological characteristics, a significant difference in CSV-CTC count was found among patients at different Ras gene status and with the primary tumor arising in different sites (P = 0.03, P < 0.01) (Table 2). CSV-CTC counts had no correlation with patient gender, age >  = 60 years, the N-stage, t the T-stage, the BRAF genotype status, and the carcinoembryonic antigen (CEA) level (p > 0.05, Table 2). As for the other two CTC subtypes (PanCK-CTC and Mixed-CTC), apart from a significantly higher count of PanCK-CTCs observed in stage IV CRC patients (p < 0.05), no significant association was found between them and clinicopathologic features (p > 0.05) (Table 2). We examined the proportion of CSV-CTCs relative to the total CTCs in each patient. The findings revealed that on average, Stage IV patients had 62.8% ± 15.9% CSV-CTCs, whereas Stage III patients had 51.3% ± 21.5%. This indicates a statistically significant difference between the two groups (Fig. 1C). Since high-grade microsatellite instability (MSI-H) was detected in the tissue specimens from only two patients, their CTC counts were not compared with those of patients with microsatellite stability (MSS).

**Table 2:** Correlation between various CTC phenotypes counts and clinicopathological characteristics.

Characteristic		Total CTC (x ± s)	p-value	CSV-CTC (x ± s)	p-Value	Panck-CTC (x ± s)	p-value	Mixed-CTC (x ± s)	p-value
Age	< 60	3.9 ± 2.6	0.73	2.1 ± 1.7	0.48	0.7 ± 0.8	0.50	1.0 ± 0.8	0.33
	≥ 60	3.6 ± 2.5		2.5 ± 1.9		0.6 ± 0.6		0.8 ± 0.7	
Gender	Male	4.3 ± 2.6	0.22	2.6 ± 1.9	0.23	0.8 ± 0.7	0.28	0.8 ± 0.6	0.86
	Female	3.6 ± 2.4		2.1 ± 1.8		0.6 ± 0.6		0.9 ± 0.8	
T-stage	T1-2	1.3 ± 2.3	0.07	0.7 ± 1.2	0.11	0.3 ± 0.6	0.36	0.3 ± 0.6	0.20
	T3-4	4.0 ± 2.5		2.4 ± 1.8		0.7 ± 0.7		0.9 ± 0.7	
N-stage	N1	3.4 ± 2.6	0.14	2.0 ± 1.9	0.12	0.6 ± 0.6	0.28	0.8 ± 0.7	0.50
	N2	4.2 ± 2.4		2.6 ± 1.8		0.7 ± 0.7		0.9 ± 0.7	
M-stage	M0	3.3 ± 2.4	< 0.01*	1.9 ± 1.8	< 0.01*	0.6 ± 0.6	0.02*	0.8 ± 0.7	0.06
	M1a	5.3 ± 2.2		3.4 ± 1.5		0.9 ± 0.7		1.1 ± 0.8	
CEA level	normal	3.6 ± 2.3	0.40	2.3 ± 1.7	0.65	0.6 ± 0.7	0.47	0.7 ± 0.7	0.18
	Abnormal	4.1 ± 2.7		2.4 ± 2.0		0.7 ± 0.6		1.0 ± 0.7	
Tumor location	Right	5.4 ± 2.3	< 0.01*	3.5 ± 1.9	< 0.01*	0.9 ± 0.6	0.08	1.1 ± 0.7	0.10
	Left	3.2 ± 2.3		1.8 ± 1.6		0.7 ± 0.6		0.8 ± 0.7	
BRAF gene status	Wild	3.9 ± 2.4	0.77	2.3 ± 1.7	0.87	0.7 ± 0.7	0.86	0.9 ± 0.7	0.98
	Mutation	4.1 ± 3.1		2.4 ± 2.3		0.6 ± 0.6		0.9 ± 0.7	
RAS gene status	Wild	3.4 ± 2.4	0.03*	2.0 ± 1.8	0.03*	0.6 ± 0.6	0.38	0.8 ± 0.7	0.24
	Mutation	4.6 ± 2.6		2.9 ± 1.7		0.8 ± 0.7		1.0 ± 0.7	

*Represents a statistically significant difference.

### Evaluating the influence of three CTC phenotypes on DFS in CRC patients over a two-year period

**Figure 2:**
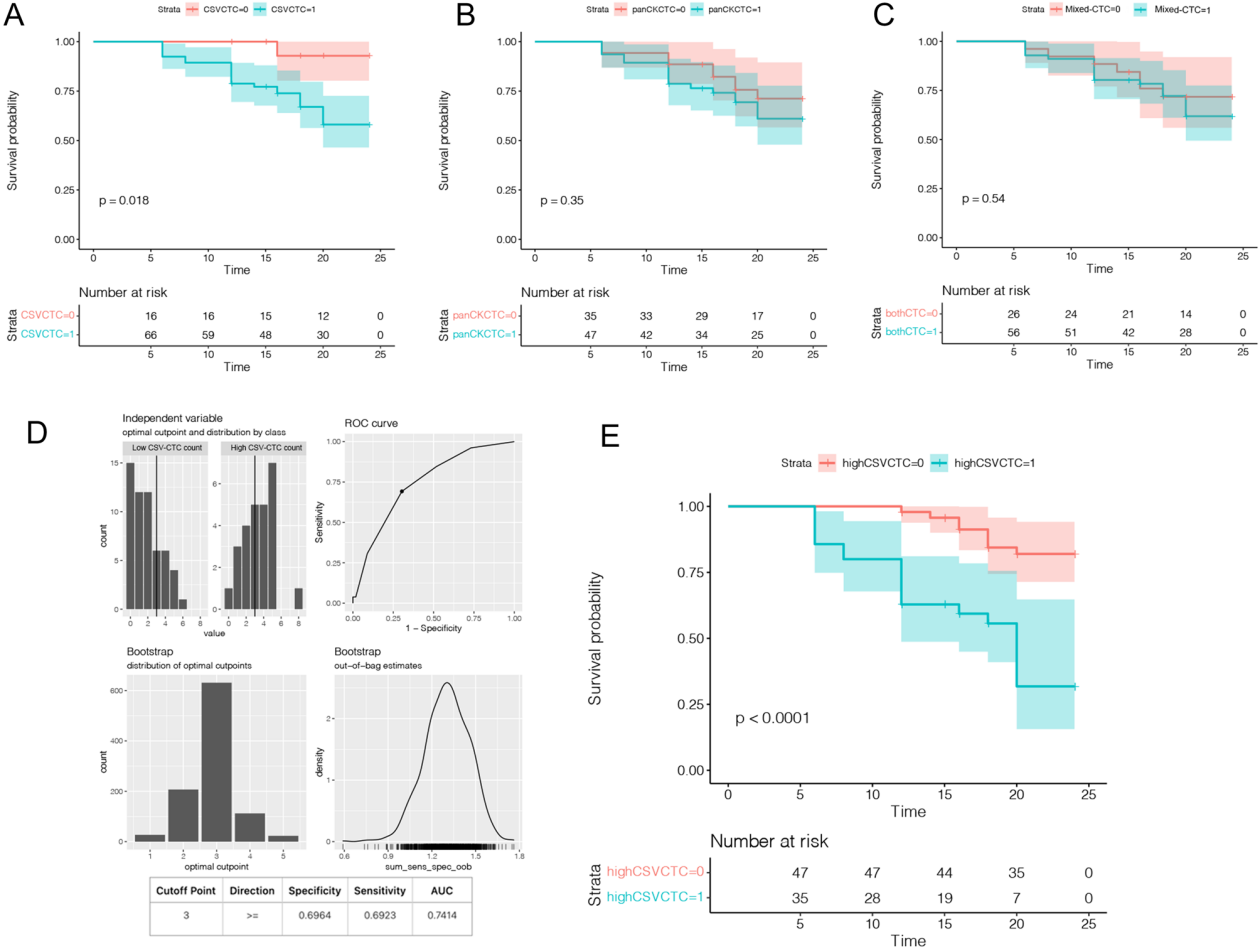
The prognostic role of CTC phenotypes. (**A**) Kaplan–Meier curve of DFS according to CSV-CTC counts. (**B**) Kaplan–Meier curve of DFS according to PanCK-CTC counts. (**C**) Kaplan–Meier curve of DFS according to Mixed-CTC counts. (**D**) A ROC curve for determining the best cut-off value for the CSV-CTC count; 0: Low CTC Count; 1: High CTC Count. (**E**) Kaplan–Meier curve of DFS in CRC patients according to the cut-off value of the CSV-CTC count.

Of the 82 CRC patients enrolled, 65 patients had CSV-CTCs detected, 47 patients had PanCK-CTCs detected, and 56 patients had Mixed-CTCs detected. Except for 9 patients, all the other patients completed the follow-up. DFS was used as the follow-up index. Twenty-seven patients had progressive disease, and the most fast disease progression was observed in the sixth month. Their two-year DFS was 67.1%. Kaplan–Meier survival analysis showed that the DFS of the patients with CSV-CTCs detected was 19.8 ± 0.8 weeks (95% CI 18.2–21.1 weeks), whereas that of the patients without CSV-CTCs detected was 23.4 ± 0.6 weeks (95% CI 22.3–24.5 weeks). A comparison of the DFS between the two groups of patients indicated that the group with CSV-CTCs detected had a worse DFS (HR = 3.78, 95% CI 1.55 to 9.26, p = 0.04) (Fig. 2A). However, there were no significant differences in DFS between the two other CTC phenotypes (P = 0.18, HR = 1.65, 95% CI 0.82 to 3.31 for the PanCK-CTC group, and P = 0.22, HR = 1.52, 95%CI 0.78 to 2.97 for the Mix-CTC group) (Fig. 2B and C). As shown in Fig. 2D, a ROC curve was drawn to determine the cut-off value of the CSV-CTC count that would provide the most accurate prediction result. When the cut-off value of the CTC count was set to 3 to distinguish between high and low CTC counts, the sensitivity and specificity were 0.70 and 0.69, respectively (Fig. 2D), with a maximum AUC of 0.74. Based on the cut-off values, patients were divided into groups with high and low CSV-CTC counts. The DFS of patients with high CSV-CTC counts was significantly shorter than that of patients with low CSV-CTC counts (HR = 4.678, 95% CI  2.059 to 10.63, P < 0.001) (Fig. 2E).

### Univariate and multivariate analyses for identifying risk factors associated with a shorter DFS

In order to identify the factors influencing 2-year DFS in the 82 CRC patients (Table 3), a comprehensive analysis was conducted. The univariate Cox regression analysis revealed statistically significant associations between DFS and several clinical parameters, including the advanced T-stage, the N-stage, clinically resectable distant metastases (M1 stage), tumor location in the right colon, RAS gene mutation, presence of CSV-CTCs, and high CSV-CTC counts (all with p < 0.05). Subsequently, a multivariate Cox regression analysis was performed for adjusting the aforementioned factors. The results demonstrated that high CSV-CTC counts ((HR = 1.47, 95% CI 1.12–1.92; p < 0.01) and stage N2 (HR = 2.13, 95% CI 1.01–4.50; p < 0.05) were significantly associated with a shorter 2-year DFS.

**Table 3:** Risk factors associated with disease progression within 2 years.

Variable	Univariate HR (95%CI)	P value	Multivariate HR (95%CI)	P value
Age (years)				
< 60	Reference	0.44		
≥ 60	1.01 (0.98–1.05)			
Gender				
Male	Reference	0.21		
Female	0.61 (0.28–1.32)			
T stage				
T1		0.02		0.65
T2	Reference		Reference	
T3/4	3.16 (1.24–8.06)		1.26 (0.47–3.39)	
N stage				
N1	Reference	0.02	Reference	< 0.05
N2	2.16 (1.16–4.04)		2.13 (1.01–4.50)	
M stage				
M0	Reference	< 0.01	Reference	0.09
M1	5.20 (2.34–11.43)		2.11 (0.90–4.96)	
Neochemotheraphy				
No	Reference	0.30		
Yes	1.89 (0.57–6.31)			
CEA				
Normal	Reference	0.59		
Abnormal	1.24 (0.57–2.70)			
BRAF gene status				
Wild	Reference	0.15		
Mutation	0.34 (0.08–1.45)			
Tumor location				
Left side	Reference	0.01	Reference	0.21
Right side	2.76 (1.28–5.97)		1.67 (0.74–3.76)	
RAS gene status				
Wild	Reference	< 0.01	Reference	0.17
Mutation	3.89 (1.73–8.76)		1.88 (0.76–4.64)	
Mixed-CTC presence				
Yes	Reference	0.14		
No	2.11 (1.07–3.12)			
PanCK-CTC presence				
Yes	Reference	0.11		
No	1.63 (0.64–3.03)			
CSV-CTC presence				
Yes	Reference	0.02	Reference	0.13
No	1.36 (1.16–1.58)		1.13 (0.81–1.81)	
CSV-CTC count ≥ 3				
No	Reference	< 0.01	Reference	< 0.01
Yes	5.26 (2.24–12.37)		1.47 (1.12–1.92)	

### Nomogram creation and comparison

**Figure 3:**
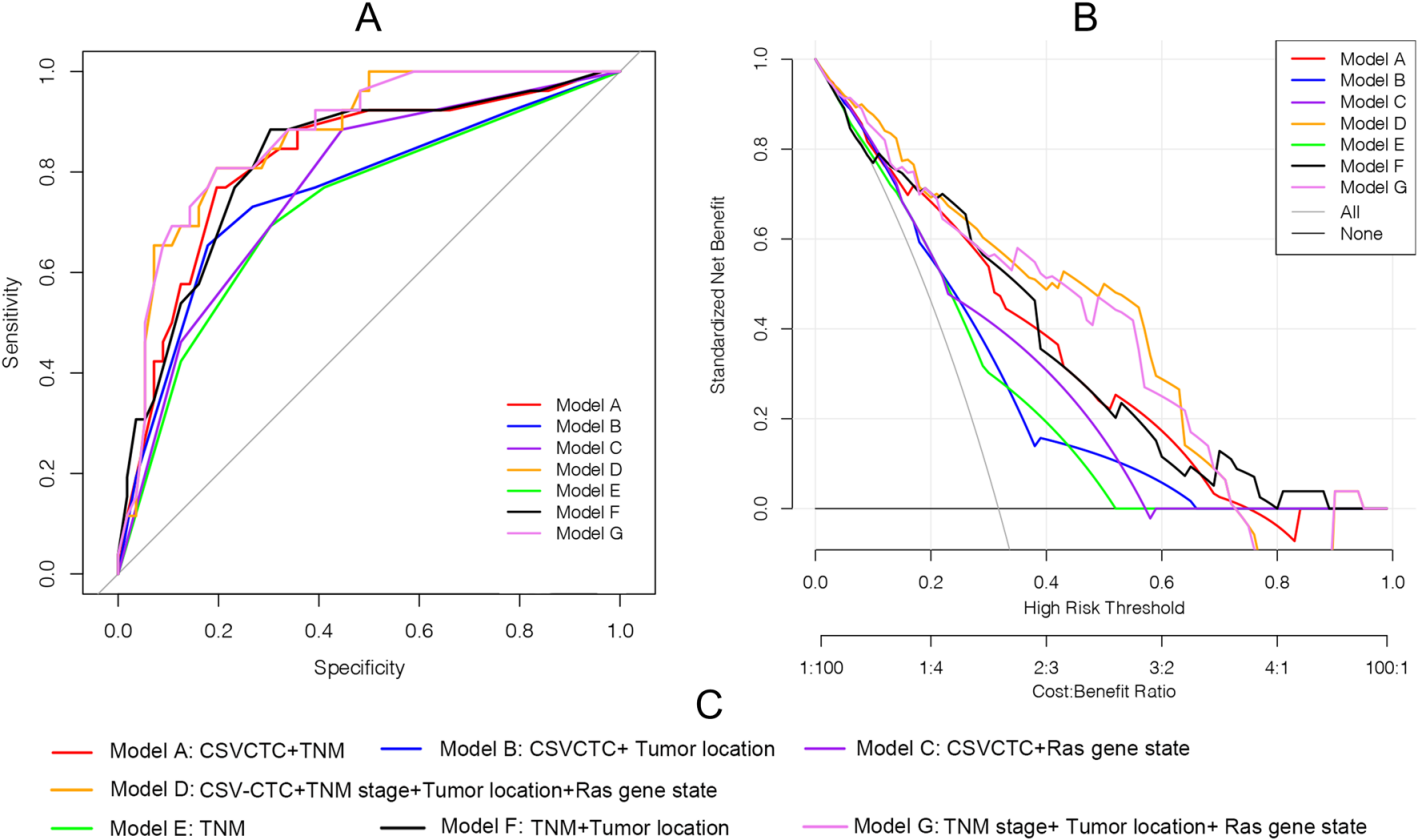
Comparison of different compound models. (**A**) ROC curves for evaluating the predictive accuracy of the models. (**B**) DCA curves for evaluating the clinical benefit of the models. (**C**) A detailed description of the compound models.

**Figure 4:**
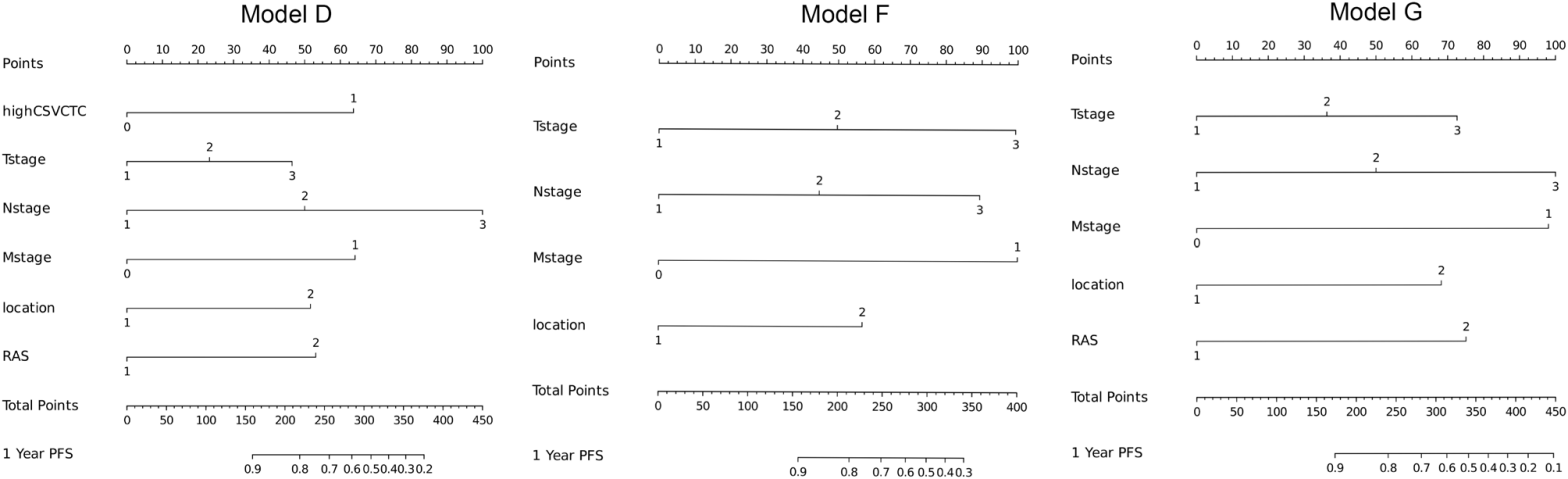
Nomograms of the three best models.

Compound models integrating features that significantly influenced the prognosis of patients based on the univariate analysis were constructed. The combinations included Model A: CSV-CTC + TNM stages; Model B: CSV-CTC + Tumor location; Model C: CSV-CTC + Ras gene state; Model D: CSV-CTC + TNM stages + Tumor location + Ras gene state; Model E: TNM stages; Model F: TNM stages + Tumor location; and Model G: TNM stages + Tumor location + Ras gene state. ROC curves comparing the models were plotted (Fig. 3A). Model D performed the best, with an AUC of 0.877, followed by Model G (with an AUC of 0.869) and Model A (with an AUC of 0.839) successively. Model E performed the worst. The AUCs of Models B, C, D and F were 0.771(95% CI 0.5985–0.7488), 0.752 (95% CI 0.5195–0.6684), 0.794 (95% CI 0.533–0.614) and 0.800 (95% CI 0.544–0.696) respectively. DCA was conducted to evaluate the effects of different compound models (Fig. 3B), and the analysis results showed that Model E was more effective than the other compound models within the threshold probability range of 0.2 to 0.95. Considering that Models A, E, and G had better AUC and DCA results, visual nomograms of these three compound models (Fig. 4) were constructed to facilitate clinical application.

## Discussion

EMT endows CTCs with mesenchymal phenotypes, and is an early event in the metastatic process^12^. It is thus conceivable that the detection of EMT markers in CTCs may facilitate the detection of CTCs. EMT transfers vimentin from intracellular regions to the cell surface, resulting in the generation of CSV^13^. It is reported that CSV can be used as a target for capturing mesenchymal CTCs, and the rate of CTC detection by the CSV antibody is higher than that by the EpCAM antibody in breast and pancreatic cancer patients^14,15^. In comparison to our previous study^10^, although the criteria for enrolling patients were the same, the CTC analysis platform differed. Specifically, the capture antibodies, processed blood volume, detection antibodies, and collection tubes were different. Despite these differences, the detection rates for epithelial CTCs were similar: 42.1% in our previous study versus 49.7% in the present one. These findings align with the data reported in other studies^16,17^. However, the mean counts of epithelial CTCs detected in the present study (0.8 PanCK-CTCs) was smaller than that reported in other studies (including our previous study). The reason may be that some epithelial CTCs were categorized as Mixed-CTCs in the present study because of the finer classification of CTCs. In this study, CTCs were detected in 86.6% of CRC patients, and the median number of CTCs was 4(range: 0–11). Both the CTC detection rate and the number of detections were significantly higher than those in our previous study. Furthermore, the present study showed that CSV-CTCs, which have mesenchymal cell characteristics, accounted for 62.8% ± 15.9% of stage IV patients, whereas 51.3% ± 21.5% of stage III patients had a significant difference. In addition, it was also found that CTC counts were significantly associated with patients' T-stage, M-stage, and site of the primary focus, and CSV-CTCs were the main reason for this association. Ren Zhao et al. obtained the same results by performing CTC testing on 1024 CRC patients, and the expression of CTCs with mesenchymal features was significantly higher in tumors with metastasis^11^. Our previous result demonstrated that EpCAM-based detection methods might not be adequate for diagnosing CTCs. Compared with EpCAM-based strategies, the CSV-based strategy is slightly more effective. The increased CTC counts observed in patients with stage T3/4 CRC compared to those with stage T1/2 CRC was attributed to higher likelihood of vascular invasion and the longer tumor progression in stage T3/4 patients^18^. Regarding the elevated CTC counts in patients with primary tumors located in the right hemicolon, this phenomenon might be linked to the poorer biological behavior of tumors in this region. Additionally, most right hemicolon tumors were asymptomatic and distended during the early stages, leading to a more extended disease course and a progressively advancing tumor^19,20^.

CSV-based strategies for CTC detection not only have higher sensitivity but may also provide insight into the biological characterization of tumor cells. Lu et al.^21^ used the EMT-CTC testing strategy, and it was found the increased level in mesenchymal CTCs was a predictor of poor outcomes after first-line chemotherapy in early- and mid-stage CRC patients. However, few studies have examined the expression of different CTC phenotypes in later-stage CRC patients, their relationship with clinicopathological features, and their impact on patient prognosis after treatment. The results of the present study showed that the CTC count and the proportion of CSV-CTCs in CTCs in stage IV patients were significantly higher than those in stage III CRC patients. The Kaplan–Meier analysis revealed that patients with CSV-CTCs detected had a significantly shorter DFS. In contrast, epithelial and mixed CTCs did not affect patient prognosis. It appeared that mesenchymal CTCs were the prominent phenotype affecting the prognosis of patients. In patients with high CSV-CTC counts, the DFS was significantly worse than that in patients with low CSV-CTC counts (HR = 4.678, 95% CI 2.059 to 10.63, p < 0.001). To our knowledge, the present study is the first study that applied the CytoSorter^®^ platform to determine the cut-off value of the CSV-CTC count for predicting post-treatment prognosis in advanced CRC. Although different CTC test platforms employed different methodologies, the studies made the same conclusion that CSV-CTCs are predictive biomarkers for cancer, and a CSV-based strategy is slightly more effective than one based on EpCAM^14,21,22^. The findings of the current investigation conclusively show that the presence and increased levels of CTCs have a negative impact on patient prognosis. Moreover, the multivariate analysis revealed that a high CSV-CTC count was an independent risk factor for disease progression within 2 years after oxaliplatin-based first-line chemotherapy in patients with stage III/IV CRC. Therefore, setting the cut-off value of the CSV-CTC count accordingly is of clinical significance.

Despite of the clinical utility of CSV-CTCs^14,22,23^, they have not yet been fully exploited due mainly to the lack of a simple clinical assessment model. To facilitate the clinical application of the CSV-CTC test, the present study developed seven nomogram graph models using CSV-CTC data and clinicopathologic characteristics, and the models were tested through univariate and multivariate analyses. Four models were based on CSV-CTCs and clinicopathological characteristics, and the other three models only included clinicopathological factors. The results indicated that Models D and G provided the best predictions with AUC values of 0.877 and 0.839, respectively. In both models, the clinicopathological factors were consistent, with the only difference being that Model D included CSV-CTCs while Model G did not. Despite having better AUC values, Model D was not statistically different from Model G. Are CSV-CTCs of little clinical significance? Considering the correlation of CSV-CTC expression with the T-stage, the M-stage, and the site of the primary tumor (Fig. 1), we believed that CSV-CTCs played the same prognostic role as these clinicopathological factors in the compound models. Moreover, CSV-CTCs improved the AUC values and the clinical benefits of the compound models, as demonstrated by DCA curves in Fig. 3. Because CTC testing is not available in some hospitals or patients are unwilling to undergo expensive CTC testing, the prognosis can only be predicated using clinicopathological factors. Therefore, we finally constructed nomograms for Models D and G (Fig. 4). To the best of our knowledge, the present study is the first study to incorporate CSV-CTCs and clinicopathological factors into nomogram models for predicting the prognosis of advanced CRC after oxaliplatin-based first-line adjuvant chemotherapy. As a result, the CSV-CTC assay is more appropriate for clinical use, while the prognosis prediction model is more accurate.

**Figure 5:**
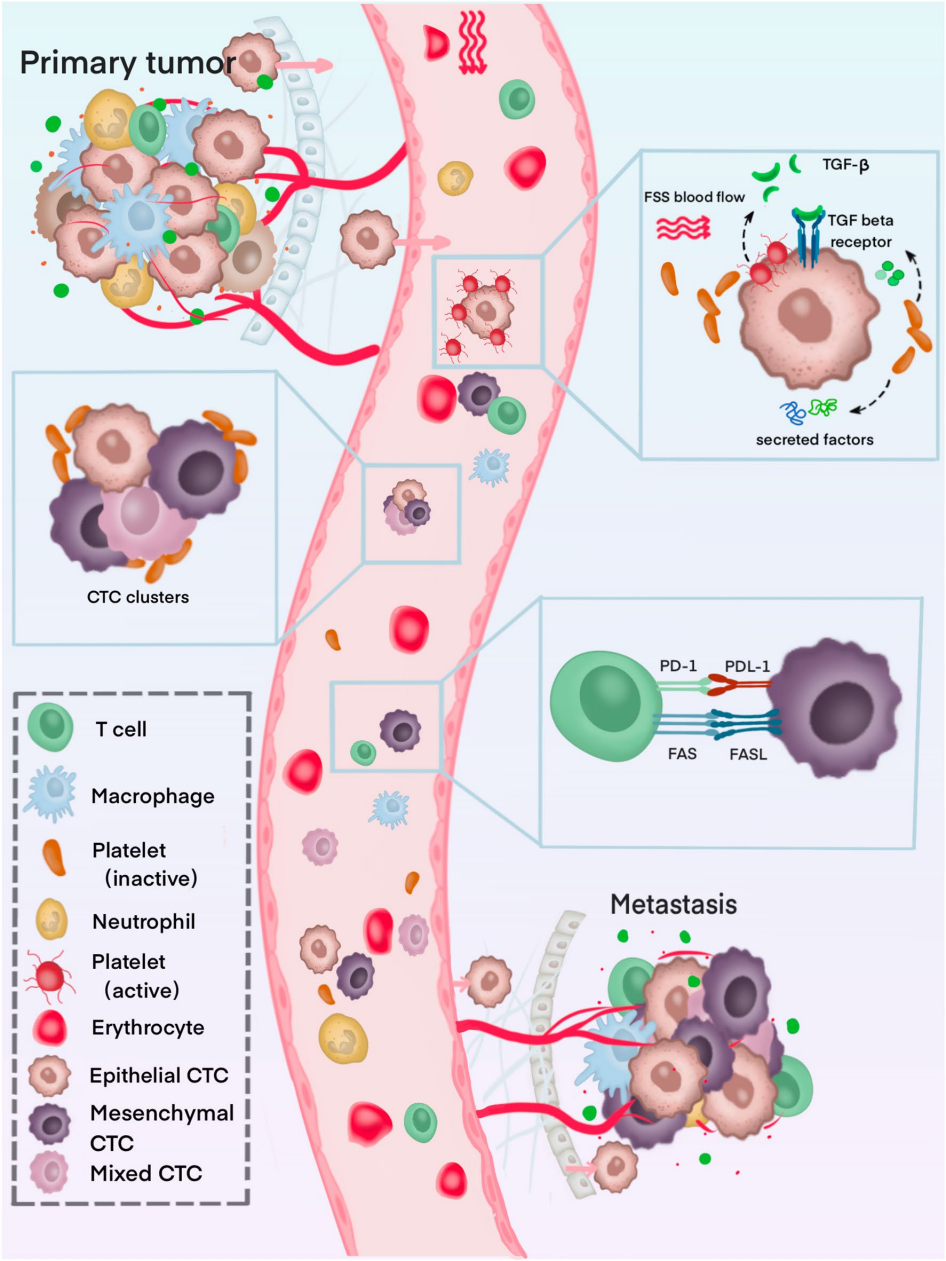
Overview of CTC-blood interactions during haematogenous dissemination.

Our results raised another two important points, namely, why do mesenchymal CTCs predominate in CTCs in the peripheral blood of CRC patients, and is this phenotype of CTC more likely to affect patient prognosis? The mechanisms might be explained by the following. Once CTCs are disseminated, they will spend a brief period before either being trapped in capillaries or being removed by the immune system. Approximately 0.1% of CTCs survive in the bloodstream for more than 24 h^24,25^. Platelets take an important role. As CTCs enter the circulatory system, they are surrounded by platelet-rich thrombin, which prevents them from being physically removed^26^. Additionally, platelets secrete a variety of growth factors during activation, including platelet-derived growth factors (PDGFs), vascular endothelial growth factors (VEGFs), and transforming growth factor beta (TGF-A)^27^. These growth factors serve as an aid to CTC growth^28,29^ as well as a means of evading apoptosis if they are exposed to chemotherapy^30^. As a result of TGF signaling, platelets induce EMT in CTCs, thereby increasing the possibility of metastasis^31,32^. The inhibition of TGF-β by platelets or the blocking of the NF-κB pathway in cancer cells reduced metastasis in vitro^32^. A further consideration is that mesenchymal CTCs tend to cluster more frequently than epithelial ones. Such clusters in circulation may benefit from this expression profile since invasion requires a mesenchymal phenotype^33^. Several cancer types have been shown to have clusters of CTCs, which indicate a poor clinical outcome^33,34^. For CTC clusters, cooperation between similar cell types shields them from shear forces, environmental stress, and immune attack. In addition, EMT plays a key role in tumor immunosuppression and immune evasion^35^. There is evidence that EMT activates immune checkpoint molecules, such as PD-L1^36,37^. Immune escape may be more likely to occur in mesenchymal CTCs, thus promoting cancer progression. Because of these mechanisms, it is believed that CTCs that have undergone EMT survive better in circulation and are more likely to metastasize and settle in distant locations. Figure 5 illustrates the mechanisms underlying the survival and metastatic potential of mesenchymal CTCs.

## Conclusions

According to the results of the current study, mesenchymal CTCs predominate among all CTCs in the peripheral blood of CRC patient. EpCAM-based detection methods may not be adequate for diagnosing CTCs in CRC patients. CSV-CTCs are positively correlated with the T-stage, the M-stage, and the location of the primary tumor. It suggests that CSV-CTCs represent a more invasive phenotype of CTCs. The CSV-based CTC detection strategy not only has high sensitivity but may also provide more accurate prognostic information. High CSV-CTC counts (CSV-CTC ≥ 3) are an independent risk factor for disease progression. Nomograms that incorporate multiple variables are proven effective in assessing personalized risk and aiding doctors in decision making.

This study has some limitations. Firstly, there was some heterogeneity in surgical outcomes of stage III CRC patients because surgery was performed by two medical groups from two different hospitals. Secondly, there was a limited number of participants, and long-term results are not available now. Thirdly, the nomogram models have not been further validated in the new dataset, and their application has not been assessed. Further multicenter and large-cohort studies are needed to verify our findings.

## Materials and methods

### Ethics and participants

Patients with CRC who underwent CTC detection at Ningbo Medical Centre Li Huili Hospital and The First Affiliated Hospital of Ningbo University between December 2019 and August 2020 were included in the study. The following inclusion criteria were applied. (a) Patients with CRC were confirmed based on pathologic reports obtained from the Pathology Center of Ningbo, where the tumor tissues were obtained through electronic colonoscopy. (b) Patients were classified at clinical stage III-IV based on preoperative CT or MRI scans following the 8th edition of the American Joint Committee on Cancer staging system^38^. (c) Patients had not received any prior treatment. (d) Patients had available data available on the status of KRAS/NRAS/BRAF gene mutation. (e) CTC detection was performed before specific treatment (radical surgery in stage III cases and neoadjuvant therapy in stage IV cases). The following exclusion criteria were applied. (a) The histologic type of the tumor was not called adenocarcinoma, and the pathological diagnosis was uncertain or questionable. (b) The tumor was evaluated clinically unresectable by the multi-disciplinary team. (c) Patients received R2 resection. (d) Patients had a history of CRC radical resection. (e) The data were incomplete. Neoadjuvant treatment was not given to any patient with stage III CRC. Surgeries were performed by two medical teams from two different hospitals. Both medical teams are highly skilled and experienced in conducting CRC surgeries. After radical resection, all patients received a six- to eight-cycle oxaliplatin-based perioperative adjuvant chemotherapy regimen. Ethics approval was obtained from the Ethics Committee of Ningbo Medical Center Li Huili Hospital (approval number: DYLL2018005) and The First Afflicted Hospital of Ningbo University (approval number: DYLL2019213), aligning with ethical standards and the principles outlined in the Helsinki Declaration. Blood and tissue samples were collected with the written consent of each patient. Patients' age, gender, tumor histology, TNM staging, serum carcinoembryonic antigen level, and location of the tumor were all collected as clinical and pathological information. Patients were followed up for a minimum of two years. The primary outcome monitored was DFS. Enhanced computed tomography and magnetic resonance imaging were used to detect tumor recurrence in the abdominal cavity.

### Identification of CTC phenotypes

We used the CSV mesenchymal CTC kit from CytoSorter^®^ (Hangzhou Watson Biotech, Hangzhou, China) for CTC detection, and details about this kit were provided in our previous study^23^. CTC detection was carried out following the manufacturer's protocol. Briefly, peripheral blood (7.5 ml) was harvested in EDTA-pretreated blood collection tubes. The blood samples were processed within 6 h after collection. A density gradient centrifugation method was used to obtain peripheral blood mononuclear cells (PBMCs). Subsequently, the PBMC sample solution was transferred to the spiral sample tube on CytoSorter^®^. Before we placed the streptavidin-functionalized CytoChipNano onto CytoSorter^®^, a biotin-labeled CSV antibody was applied. CytoSorter^®^ software provided independent control over the enrichment procedure for each CTC-capturing antibody. The CytoChipNano was then removed from the CytoSorter^®^ after CTC enrichment, and the cells were stained with CSV-fluorescein isothiocyanate (CSV-FITC) and PanCK. In addition, lymphocyte antigen-phycoerythrin (CD45-PE) was used to label white blood cells, while the nucleus was stained with Hoechst. Two experienced technicians used an OPPNO immunofluorescence microscope (DSY5000X, OPPNO, Chongqing, China) to identify CTC phenotypes. Mesenchymal CTCs were CSV-FITC-positive, CD45-PE-negative, and PanCK-negative, and appeared green and blue but not orange under a fluorescent microscope. Epithelial CTCs were positive for PanCK and negative for CSV-FITC and CD45-PE, showing a red or orange appearance under the fluorescent microscope rather than green or blue. In cases negative for CD45-PE and positive for CSV-FITC and PanCK, mixed CTCs were detected. The morphology of all identified cells was examined under bright-field illumination.

### Statistical analysis

The statistical analysis was performed using R studio 4.0.4 (The R Foundation for Statistical Computing), Blue4ML version 0.0.1 (Ningbo Catalyst Technology Co. Ltd) and Prism 5.0 (GraphPad Software Inc., San Diego, CA, USA). A two-sided P < 0.05 indicated statistically significant differences. For the univariate analysis, independent t-tests or Mann–Whitney U tests were employed to assess differences between patient groups based on continuous variables. Fisher's exact test or chi-square test was utilized for the analysis of categorical variables. Analysis of variance (ANOVA) and the Kruskal–Wallis H test were conducted for comparing three or more groups.

### Consent to participate

Informed consent was obtained from all subjects and/or their legal guardian(s).

## Data Availability

The datasets analyzed during the current study are available from the corresponding author on reasonable request.
