# Author Correction: Evaluation of cell surface vimentin positive circulating tumor cells as a prognostic biomarker for stage III/IV colorectal cancer

**DOI:** 10.1038/s41598-024-56483-7

**Published:** 2024-03-18

**Authors:** Jiazi Yu, Mian Yang, Tao Peng, Yelei Liu, Yuepeng Cao

**Affiliations:** 1 Department of Colorectal Surgery, Ningbo Medical Centre Li Huili Hospital, Ningbo, People’s Republic of China; 2 Department of General Surgery, Ningbo Medical Treatment Centre Li Huili Hospital, 1111 Jiangnan Road, Ningbo, 315000 People’s Republic of China; 3 Department of Colorectal Surgery, The First Affiliated Hospital of Ningbo University, Ningbo, People’s Republic of China

Correction to: *Scientific Reports* 10.1038/s41598-023-45951-1, published online 01 November 2023

The original version of this Article contained an error in the order of the author names, which was incorrectly given as Yuepeng Cao, Mian Yang, Tao Peng, Yelei Liu & Jiazi Yu.

As a result, Jiazi Yu was incorrectly listed as a corresponding author. The correct corresponding author for this Article is Yuepeng Cao. Correspondence and request for materials should be addressed to cao_yuepeng@163.com

Additionally, the statement of Equal Contribution contained errors due to the incorrect order of author names.

“These authors contributed equally: Yuepeng Cao and Mian Yang.”

now reads:

“These authors contributed equally: Jiazi Yu and Mian Yang.”

Lastly, the Author Contributions section was corrected. It now reads:

“J.Y.: Conceptualization, Data curation, Investigation, Methodology, Project administration, Supervision, Visualization, Writing—original draft; M.Y.: Conceptualization, Investigation, Methodology, Supervision, Validation; T.P.: Data curation, Formal analysis, Investigation; Y.L.: Data curation, Formal analysis, Writing—original draft; Y.C.: Conceptualization, Data curation, Formal analysis, Funding acquisition, Investigation, Writing—original draft. All authors read and approved the final manuscript.”

The original Article has been corrected.

